# Psychological Outcomes After Mastectomy With vs Without Nipple–Areolar Complex Reconstruction: A Propensity-Matched Cohort Study of 5160 Patients

**DOI:** 10.1093/asjof/ojaf161

**Published:** 2025-11-29

**Authors:** Yousef Tanas, Philong Nguyen, Joshua Wang, Stephen Chen, Sarya Swed, Aldona Spiegel

## Abstract

**Background:**

Breast reconstruction after mastectomy restores both physical and emotional well-being. Nipple–areolar complex (NAC) reconstruction represents the final stage of restoration, yet its psychological impact remains unclear.

**Objectives:**

This study evaluates mental health outcomes following NAC vs non-NAC breast reconstruction.

**Methods:**

The TriNetX Research Network was queried for female breast cancer patients ≥ 18 years who underwent mastectomy with subsequent breast reconstruction from 2003 to 2023. Cohorts were stratified by NAC reconstruction. Propensity score matching was performed for demographics, comorbidities, substance use, cancer treatment, and pre-existing psychiatric conditions. Outcomes included various psychological conditions such as anxiety, depression, and antidepressant use at 3-, 12-, and 24-months postoperatively. Risk ratios (RRs), 95% CIs, and *P* values were calculated.

**Results:**

Nipple–areolar complex reconstruction was associated with significantly lower risks of several psychiatric outcomes. Anxiety was reduced at 3 months (RR = 0.40; *P* = .0004), 12 months (RR = 0.62; *P* = .0009), and 24 months (RR = 0.79; *P* = .026). Adjustment disorder was less frequent at 12 (RR = 0.52; *P* = .0099) and 24 months (RR = 0.65; *P* = .024). Substance use disorder (RR = 0.40; *P* < .0001) and antidepressant use (RR = 0.82; *P* = .034) were also significantly reduced at 24 months.

**Conclusions:**

Nipple–areolar complex reconstruction is linked to lower long-term risks of various psychological conditions following mastectomy, suggesting psychosocial benefits beyond aesthetic restoration. These findings support holistic consideration of both surgical and mental health factors in reconstructive counseling.

**Level of Evidence: 3 (Risk):**

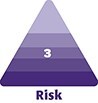

Breast cancer is the most frequently diagnosed cancer among women, with approximately 1 in 10 women affected each year. Treatment often includes surgical oncologic resection, such as mastectomy, followed by breast reconstruction for those who desire it. Preserving or reconstructing the nipple–areolar complex (NAC) is central to cosmetic satisfaction after mastectomy; nipple-sparing strategies are increasingly used to maintain contour and body image.^1^ In cases where a nipple-sparing approach is not feasible, or when complications arise during prior reconstruction, the NAC may be absent from the reconstructed breast. For patients who elect to restore the NAC, this step is typically performed as the final stage of the reconstructive process. The goal of NAC reconstruction is to recreate both the nipple and areola, achieving symmetry in position, size, shape, texture, pigmentation, and projection relative to the contralateral breast.^2-7^

Nipple–areolar complex reconstruction can be achieved through several techniques, including nipple grafting, local tissue flaps, and medical tattooing. Prior research has demonstrated an association between higher levels of patient satisfaction with breast reconstruction and the presence of a nipple and areola, mitigating the psychological distress associated with a breast cancer diagnosis. This aspect of reconstruction plays an important role in helping patients reclaim a sense of bodily integrity and integrate the reconstructed breast into their self-image. Nevertheless, not all patients pursue NAC reconstruction, and the decision remains a personal one based on individual preferences, expectations, and recovery goals.^8-11^

While the psychological benefits of NAC reconstruction in improving patient satisfaction and body image are well recognized, its potential impact on mental health outcomes remains largely unexplored. To date, there is currently no literature evaluating how NAC reconstruction influences the incidence of various psychological conditions such as anxiety, depression, post-traumatic stress disorder (PTSD), and substance use disorders among breast cancer survivors. This represents a critical gap, as many patients face substantial psychosocial challenges throughout and beyond their treatment. A better understanding of these associations is essential to support informed decision-making and to ensure that reconstructive care addresses not only physical restoration but also long-term psychological recovery.^12,13^

To our knowledge, this is the first study to directly address this gap by leveraging a large, multi-institutional database to evaluate the incidence of psychological conditions in patients who undergo NAC reconstruction compared to those who do not following mastectomy. By examining mental health outcomes, we aim to provide a more comprehensive understanding of the psychological trajectories associated with NAC reconstruction to improve patient counseling, support shared decision-making, and highlight opportunities for targeted psychosocial interventions throughout the reconstructive process.

## METHODS

### Data Source

**Figure 1.**
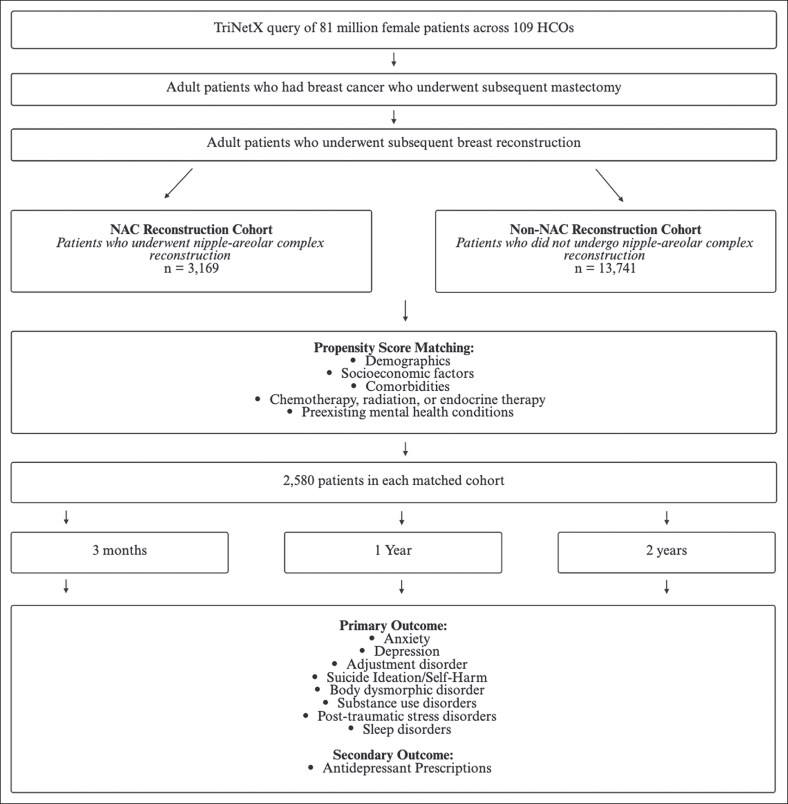
Cohort selection and data collection process.

The TriNetX Research Network is a large, federated database that includes 109 healthcare organizations (HCOs) across the United States, representing over 158 million patients. For this study, data were collected from 81 million female patients across 109 HCOs between January 1, 2003, and January 1, 2023. We identified female patients ages 18 years and older who were diagnosed with breast cancer and subsequently underwent complete mastectomy followed by breast reconstruction. The study was exempted from IRB review due to its use of de-identified patient data. Figure 1 illustrates the cohort selection process and data collection methodology for this study.

### Cohorts

Two distinct cohorts were established. The NAC cohort consisted of patients who underwent NAC reconstruction. The cohort without NAC included patients who did not undergo NAC reconstruction.

### Measured Outcomes

Primary outcomes included various psychological conditions including anxiety, depression, adjustment disorders, sleep disorders, substance use disorders, body dysmorphic disorder, PTSD, suicide ideation/self-harm, and the use of antidepressant medications. All outcomes were assessed at 3-, 12-, and 24-month intervals to evaluate the longitudinal impact of NAC reconstruction on psychological health.

To accurately assess incidence, patients with any of the specified outcomes documented prior to the observation period were excluded. Definitions for all cohorts and outcomes, along with the corresponding ICD-10, CPT, and ATC codes used in the analysis, are provided in Table 1. To minimize immortal time bias, the index date for both cohorts was consistently defined as the time of breast reconstruction.

**Table 1.** Cohort Design and Outcome Codes

Description	Codes
Cohort design	
Breast cancer	ICD-10: C50
Mastectomy	CPT: 19303; CPT: 19307; CPT: 19305; CPT: 19306
Breast reconstruction	CPT: 1014573; CPT: 1036219; CPT: 19340; CPT: 19342; CPT: 19357; CPT: 19361; CPT: 19364; CPT: 19366; CPT: 19369; CPT: 19325
Nipple–areolar Complex reconstruction	CPT: 19350
Primary outcome: psychological conditions	
Anxiety	ICD-10: F40-F48
Depression	ICD-10: F32; F32.A; F33
Sleep disorder	G47
PTSD	F43.10; F43.11; F43.12
SUD	F10-F19
Suicide attempt/self-harm	T14.91; T14.91XA; T14.91XD; T14.91XS; X71-X83; R45.851
Body dysmorphic disorder	ICD-10: F45.22
Adjustment disorder	ICD-10: F43.2
Sleep disorders	ICD-10: G47
Secondary outcome: antidepressant use	
Antidepressant prescriptions	ATC: N06A

PTSD, post-traumatic stress disorder.

### Propensity Score Matching

Propensity score matching was performed in a 1:1 ratio between NAC and non-NAC reconstruction cohorts to minimize confounding and ensure balanced baseline characteristics. Covariates for matching were identified using ICD-10, CPT, and TriNetX Curated codes, encompassing demographic, clinical, and treatment-related factors. Demographic variables included age, sex, race, ethnicity, and socioeconomic status indicators. Clinical covariates comprised BMI (categorized per World Health Organization guidelines) and comorbidities such as diabetes, cardiovascular disease, hypertension, chronic kidney disease, liver disease, and chronic obstructive pulmonary disease. Substance use factors included nicotine dependence and alcohol use disorders. Treatment-related variables accounted for receipt of chemotherapy, radiation, and endocrine therapy. To control for baseline psychological health, matching incorporated pre-existing diagnoses of anxiety, dissociative and stress-related disorders, somatoform and nonpsychotic mental disorders, depression, PTSD, substance use disorders, body dysmorphic disorder, and antidepressant use. Patients were additionally matched by the type of breast reconstruction (alloplastic vs autologous) and the type of mastectomy (simple vs radical).

A detailed comparison of demographic characteristics and covariates between the NAC and without NAC is presented in Table 2. Before matching, significant differences were observed between the groups in age, sex, race, comorbidities, oncologic treatments, and pre-existing mental health conditions (*P* < .05). Following propensity score matching, these variables were well balanced, with no statistically significant differences remaining (*P* > .05), indicating that confounding factors were effectively controlled.

**Table 2.** NAC Reconstruction vs Without NAC Reconstruction Demographics and Covariates: Pre- and Post-Propensity Score Matching

Category	Variable	Before—cohort 1 (%) *N* = 3169	Before—cohort 2 (%) *N* = 13,741	*P* value	Std diff	After—cohort 1 (%) *N* = 2580	After—cohort 2 (%) *N* = 2580	*P*-value	std diff
Demographics	Age at index (mean ± SD)	51.3 ± 10.1 (3168/100%)	51.1 ± 11.2 (13,737/100%)	.3255	0.0200	51.4 ± 10.1 (2580/100%)	51.4 ± 11.2 (2580/100%)	.9461	0.0019
	Female	3168/100%	13,737/100%	—	—	2580/100%	2580/100%	—	—
Race/ethnicity	Not Hispanic or Latino	2527/79.77	11,300/82.26	.0010	0.0636	2081/80.66	2058/79.77	.4216	0.0224
	White	2303/72.67	10,590/77.09	<.0001	0.1015	1910/74.03	1918/74.34	.7991	0.0071
	Black/African American	389/12.28	1268/9.23	<.0001	0.0985	304/11.78	302/11.71	.9311	0.0024
	Hispanic or Latino	396/12.50	1096/7.98	<.0001	0.1496	293/11.36	289/11.20	.8603	0.0049
	Asian	124/3.91	606/4.41	.2145	0.0249	101/3.92	101/3.92	1.0000	<0.0001
	Native Hawaiian/Pacific Islander	15/0.47	73/0.53	.6830	0.0082	14/0.54	16/0.62	.7142	0.0102
	American Indian/Alaska Native	10/0.32	36/0.26	.6017	0.0100	10/0.39	10/0.39	1.0000	<0.0001
Diagnoses	Hypertensive diseases	917/28.95	3552/25.86	.0004	0.0664	739/28.64	757/29.34	.5808	0.0126
	Anxiety disorders	773/24.41	3411/24.83	.6128	0.0090	632/24.95	660/25.58	.3683	0.0143
	Depressive episode	564/17.80	2203/16.04	.0154	0.0460	455/17.64	460/17.83	.8554	0.0045
	Personal hx of nicotine dependence	476/15.03	2386/17.37	.0015	0.0628	380/14.73	445/17.25	.0136	0.0694
	Sleep disorders	375/11.89	1450/10.55	.0361	0.0418	296/11.47	307/11.89	.6336	0.0124
	Type 2 diabetes mellitus	282/8.90	967/7.04	.0003	0.0642	226/8.76	226/8.76	1.0000	0.0000
Medications	Opioid analgesics	2955/93.28	12,900/93.91	.1851	0.0243	2395/92.83	2383/92.36	.5234	0.0150
	Endocrine therapies	1507/47.57	3294/23.98	<.0001	0.4874	1167/45.23	1130/43.80	.3000	0.0291
	Antidepressants	992/31.31	3807/27.71	<.0001	0.0783	809/31.36	809/31.36	1.0000	0.0000
Labs	BMI (mean ± SD)	27.7 ± 5.81 (2469/77.99)	27.4 ± 6.20 (10,617/77.29)	.0086	0.0498	27.7 ± 5.83 (1985/76.98)	27.7 ± 6.06 (2026/78.53)	.7704	0.0108
	At most 18.5 kg/m²	76/2.40	455/3.81	.0079	0.0673	70/2.71	72/2.79	.8649	0.0037
	18.5-24.9 kg/m²	1097/34.63	4856/35.35	.4429	0.0152	886/34.34	893/34.61	.8376	0.0058
	25-29.9 kg/m²	1158/36.55	3421/31.46	<.0001	0.1100	915/35.47	890/34.50	.4655	0.0211
	30-34.9 kg/m²	808/25.61	2727/19.85	<.0001	0.1407	634/24.57	599/23.22	.2532	0.0317
	35-39.9 kg/m²	333/10.51	1262/9.19	.0215	0.0445	269/10.43	273/10.58	.8559	0.0046
	≥40 kg/m²	157/4.96	723/5.26	.4827	0.0135	128/4.96	137/5.31	.5703	0.0151
Procedures (TNX curated)	chemotherapy	1479/46.69	5150/37.49	<.0001	0.1871	1160/44.61	1134/43.93	.4664	0.0137
	Radiation	191/6.03	981/7.14	.0263	0.0448	161/6.24	171/6.63	.5705	0.0158
Procedures (CPT)	19,303 Mastectomy	1370/43.25	11,754/85.55	<.0001	0.9848	1315/50.97	1371/53.14	.1186	0.0435
	19,357 Tissue expander	1309/41.32	10,828/78.82	<.0001	0.8290	1184/45.89	1141/44.23	.2289	0.0335
	19,342 Implant insertion	1212/38.26	1233/8.98	<.0001	0.7344	816/31.63	881/34.15	.0541	0.0536
	1,036,219 Breast reconstruction	898/28.35	1609/11.71	<.0001	0.4249	698/27.05	758/29.38	.0635	0.0517
	19,340 Port insertion	674/21.28	1883/13.71	<.0001	0.2002	564/21.86	607/23.53	.1530	0.0398
	19,364 Breast reconstruction	600/18.94	936/6.81	<.0001	0.3681	475/18.41	518/20.08	.1289	0.0423
	19,307 Mastectomy	240/7.58	2025/14.72	<.0001	0.2283	226/8.76	289/11.20	.0034	0.0815
	19,361 Breast reconstruction	279/8.81	615/4.48	<.0001	0.1745	207/8.02	222/8.61	.4495	0.0211
	19,366 Breast reconstruction	205/6.47	252/1.83	<.0001	0.2340	150/5.81	159/6.16	.5975	0.0147
	19,325 Breast augmentation	240/7.58	148/1.08	<.0001	0.3236	135/5.28	118/4.57	.2731	0.0328
	1,014,573 Breast reconstruction	58/1.83	94/0.68	<.0001	0.1031	46/1.78	46/1.78	1.0000	0.0000
	19,369 Breast reconstruction	10/0.32	10/0.07	.0003	0.0552	10/0.39	10/0.39	1.0000	0.0000
	19,305 Mastectomy	10/0.32	43/0.31	.9809	0.0005	10/0.39	10/0.39	1.0000	0.0000
	19,306 Mastectomy	0/0.00	10/0.07	.1287	0.0382	0/0.00	0/0.00	—	0.0000

NAC, nipple–areolar complex; SD, standard deviation.

All variables used for matching were based on diagnoses recorded within 1 year prior to the index date to control for recent health status and ensure comparable baseline characteristics across patients. This comprehensive matching strategy enabled a more robust analysis of the relationship between NAC reconstruction status and psychological outcomes.

### Statistical Analysis

Statistical analyses were conducted using the TriNetX Research Platform (TriNetX, LLC, Cambridge, MA), which integrates programming languages such as Java, R, and Python for large-scale data processing and statistical computation. One-to-one propensity score matching was performed using a greedy nearest-neighbor algorithm with a caliper of 0.1 pooled standard deviations to achieve balanced baseline characteristics between NAC and non-NAC reconstruction cohorts. Matching incorporated demographic, clinical, psychosocial, and treatment-related variables to minimize confounding. Following matching, comparative analyses were performed to assess associations between NAC reconstruction and postoperative psychiatric outcomes, including anxiety, depression, adjustment disorder, sleep disorder, substance use disorder, PTSD, body dysmorphic disorder, and antidepressant use. Relative risk ratios (RRs) with 95% CIs, hazard ratios (HRs), and corresponding *P* values were computed at 3-, 12-, and 24-month postoperative intervals. Patients with pre-existing diagnoses of the studied outcomes prior to each interval were excluded to ensure accurate incidence assessment. Statistical significance was defined as a 2-tailed *P* < .05.

## RESULTS

Before propensity score matching, the NAC reconstruction cohort included 3168 patients with a mean age of 51.3 ± 10.1 years, while the without NAC cohort included 13,737 patients with a mean age of 51.1 ± 11.2 years. After matching, both cohorts comprised 2580 patients each, with nearly identical mean ages (51.4 ± 10.1 vs 51.4 ± 11.2 years, *P* = .9461, standardized difference = 0.0019). Following matching, demographic distributions were well balanced across ethnicity and race, including proportions of White (74.0% vs 74.3%), Black or African American (11.8% vs 11.7%), Hispanic or Latino (11.4% vs 11.2%), Asian (3.9% vs 3.9%), and other racial groups (<1% each), all with nonsignificant *P* values and standardized differences < 0.03. Outcome data at 3-, 12-, and 24-month intervals are summarized in Table 3.

**Table 3.** Psychiatric and Medication Outcomes in NAC Reconstruction vs Without NAC Reconstruction Cohorts

Outcome	Interval	Cumulative incidence—with NAC reconstruction	Cumulative incidence—without NAC reconstruction	Absolute risk difference	*P*	Risk ratio	RR 95% CI	Hazard ratio	HR 95% CI
Anxiety	3 months	1.15%	2.86%	1.687%	.0004	0.402	0.237-0.68	0.399	0.234-0.678
	1 year	4.49%	7.18%	2.639%	.0009	0.623	0.469-0.827	0.613	0.458-0.82
	2 years	8.57%	10.86%	2.23%	.0256	0.786	0.635-0.972	0.767	0.613-0.958
Depression	3 months	0.90%	1.44%	0.537%	.1204	0.623	0.341-1.139	0.62	0.338-1.137
	1 year	3.76%	4.97%	1.199%	.0661	0.753	0.555-1.02	0.749	0.549-1.022
	2 years	6.69%	7.52%	0.809%	.3215	0.888	0.702-1.123	0.879	0.689-1.122
Suicide attempt/ideation or self-harm	3 months	n/a	n/a	n/a	n/a	n/a	n/a	n/a	n/a
	1 years	n/a	n/a	n/a	n/a	n/a	n/a	n/a	n/a
	2 years	n/a	n/a	n/a	n/a	n/a	n/a	n/a	n/a
PTSD	3 months	n/a	n/a	n/a	n/a	n/a	n/a	n/a	n/a
	1 year	n/a	n/a	n/a	n/a	n/a	n/a	n/a	n/a
	2 years	0.58%	0.73%	0.157%	.4781	0.778	0.388-1.56	0.776	0.386-1.561
Sleep disorders	3 months	0.97%	1.36%	0.384%	.2455	0.714	0.404-1.264	0.715	0.403-1.269
	1 year	3.40%	4.23%	0.815%	.1640	0.802	0.588-1.095	0.802	0.584-1.101
	2 years	6.68%	7.07%	0.384%	.6171	0.943	0.75-1.186	0.936	0.739-1.187
Substance use disorder	3 months	n/a	n/a	n/a	n/a	n/a	n/a	n/a	n/a
	1 year	0.74%	1.85%	1.097%	.0010	0.394	0.222-0.7	0.391	0.22-0.697
	2 years	1.45%	3.59%	2.062%	<.0001	0.402	0.266-0.606	0.395	0.261-0.6
Adjustment disorder	3 months	n/a	n/a	n/a	n/a	n/a	n/a	n/a	n/a
	1 year	0.94%	1.81%	0.854%	.0099	0.517	0.31-0.862	0.517	0.309-0.864
	2 years	1.87%	2.87%	0.968%	.0240	0.649	0.444-0.947	0.645	0.44-0.945
Body dysmorphic disorder	3 months	n/a	n/a	n/a	n/a	n/a	n/a	n/a	n/a
	1 year	n/a	n/a	n/a	n/a	n/a	n/a	n/a	n/a
	2 years	n/a	n/a	n/a	n/a	n/a	n/a	n/a	n/a
Antidepressant use	3 months	1.90%	3.10%	1.203%	.0337	0.609	0.384-0.968	0.604	0.378-0.964
	1 year	6.69%	8.65%	1.951%	.0416	0.769	0.597-0.991	0.76	0.584-0.988
	2 years	11.90%	14.58%	2.562%	.0344	0.816	0.676-0.986	0.797	0.651-0.975

HR, hazard ratio; n/a, not applicable or not available due to <10 patients having the condition; NAC, nipple–areolar complex; PTSD, post-traumatic stress disorder; RR, risk ratio.

### 3-Month Outcomes

As shown in Table 3, patients who underwent NAC reconstruction demonstrated a significant reduction in anxiety at 3 months compared to those without NAC reconstruction (RR = 0.402; 95% CI, 0.237-0.680; *P* = .0004). Although trends toward reduced depression (RR = 0.623; 95% CI, 0.341-1.139; *P* = .1204) and sleep disorders (RR = 0.714; 95% CI, 0.404-1.264; *P* = .2455) were observed, these differences did not reach statistical significance. Rates of adjustment disorder and substance use disorder were not reported at this interval. Nipple–areolar complex reconstruction was, however, associated with significantly lower antidepressant use (RR = 0.609; 95% CI, 0.384-0.968; *P* = .0337), indicating early improvements in postoperative psychosocial adaptation.

### 12-Month Outcomes

At 12 months, the protective association of NAC reconstruction persisted for anxiety (RR = 0.623; 95% CI, 0.469-0.827; *P* = .0009) and adjustment disorder (RR = 0.517; 95% CI, 0.310-0.862; *P* = .0099). While reductions in depression (RR = 0.753; 95% CI, 0.555-1.020; *P* = .0661) and sleep disorders (RR = 0.802; 95% CI, 0.588-1.095; *P* = .1640) did not achieve statistical significance, both trends remained favorable. Antidepressant use was significantly lower among NAC patients (RR = 0.769; 95% CI, 0.597-0.991; *P* = .0416), suggesting sustained reductions in depressive symptom management. Substance use disorder was also significantly reduced (RR = 0.394; 95% CI, 0.222-0.700; *P* = .0010) at this interval.

### 24-Month Outcomes

By 24 months, the NAC cohort continued to exhibit lower risks of multiple psychiatric outcomes. Anxiety remained significantly reduced (RR = 0.786; 95% CI, 0.635-0.972; *P* = .0256), as did adjustment disorder (RR = 0.649; 95% CI, 0.444-0.947; *P* = .0240). Nipple–areolar complex reconstruction was also strongly associated with decreased substance use disorder (RR = 0.402; 95% CI, 0.266-0.606; *P* < .0001) and antidepressant use (RR = 0.816; 95% CI, 0.676-0.986; *P* = .0344). Depression (RR = 0.888; 95% CI, 0.702-1.123; *P* = .3215), sleep disorders (RR = 0.943; 95% CI, 0.750-1.186; *P* = .6171), and PTSD (RR = 0.778; 95% CI, 0.388-1.560; *P* = .4781) showed no significant differences.

## DISCUSSION

This retrospective cohort study demonstrates that patients who undergo NAC reconstruction following mastectomy experience significantly lower rates of anxiety, depression, adjustment disorder, sleep disorder, and antidepressant use compared to those who do not pursue NAC reconstruction. These differences were observed consistently across multiple postoperative time points up to 24 months, suggesting a persistent association between NAC reconstruction and improved psychological health outcomes.

These findings likely reflect a combination of psychosocial influences and patient selection factors. From a reconstructive standpoint, NAC reconstruction may contribute to a more complete aesthetic restoration of the breast, helping to re-establish bodily integrity and reduce distress associated with altered body image. Prior studies have shown that body image dissatisfaction and loss of feminine identity are common sources of psychological distress after mastectomy, and completion of NAC reconstruction has been associated with increased satisfaction and quality of life in survey-based studies using tools such as the BREAST-Q.^2,4,6,9^ Recent large-scale analyses show that patients who undergo NAC reconstruction after implant-based breast reconstruction report significantly higher BREAST-Q scores for sexual well-being and satisfaction with breasts compared to those who do not undergo NAC reconstruction, with clinically meaningful improvements observed at 2 years postoperatively.^2^ Similar findings are reported in autologous reconstruction cohorts, where NAC reconstruction is linked to higher satisfaction and sexual well-being scores.^6^ Psychosocial benefits are also evident, with NAC reconstruction contributing to improved self-image and reduced body image distress.^4,9^

Importantly, studies comparing nipple-sparing mastectomy (NSM) to skin-sparing mastectomy (SSM) with or without NAC reconstruction indicate that sexual well-being is highest in NSM, but SSM with NAC reconstruction achieves comparable outcomes, emphasizing the restorative impact of NAC procedures.^9^ These findings support the routine discussion of NAC reconstruction as part of shared decision-making in breast reconstruction, given its positive influence on patient-reported outcomes.^2,4,6,9^

At the same time, patients who choose to undergo NAC reconstruction may already demonstrate stronger baseline mental health. Therefore, NAC reconstruction may represent not only the final visual stage of reconstruction but also a meaningful marker of psychological readiness and recovery.

While this dual interpretation is compelling, it is important to acknowledge the role of patient self-selection. Individuals who pursue NAC reconstruction may differ in unmeasured ways, such as personal motivation, coping mechanisms, or social support, all of which can influence long-term psychological outcomes. Although rigorous propensity score matching was applied to account for comorbidities, psychiatric history, and treatment exposures, residual confounding cannot be fully excluded. As a result, NAC reconstruction may function as both a therapeutic intervention and an indicator of favorable baseline psychological health.

Furthermore, the significantly reduced risk of substance use disorders was particularly robust and sustained across the 3-year follow-up period. This could reflect that patients who elect to complete reconstruction with NAC may be more likely to engage in healthier coping mechanisms, possibly due to stronger baseline psychological well-being. In contrast, the lack of significant differences in PTSD, body dysmorphic disorder, and sleep disturbances suggests that these conditions may be less associated with the decision to pursue NAC reconstruction.

These findings have important clinical implications for the multidisciplinary care of patients undergoing breast reconstruction. While NAC reconstruction is often viewed as an elective or aesthetic component of the reconstructive process, our results suggest that the decision to pursue NAC may serve as a marker of stronger baseline psychological well-being and greater emotional readiness for recovery as evidenced by improved patient-reported outcomes and qualitative assessments in the medical literature.^2-6,10^ Clinicians should consider incorporating discussions around psychological health and reconstructive preferences into shared decision-making, particularly in the context of survivorship care planning. The consistent associations between NAC reconstruction and lower rates of anxiety, depression, substance use disorders, and antidepressant use may reflect pre-existing psychological differences among patients who choose to complete their reconstruction with NAC. Recognizing this pattern can help identify patients who may be at higher risk for adverse mental health outcomes and who could benefit from additional screening or referral to psychosocial support services. Integrating mental health considerations into reconstructive pathways and acknowledging the psychological significance of NAC reconstruction may ultimately enhance both patient satisfaction and long-term quality of life.

While this study benefits from the use of a large, multi-institutional database and employs rigorous propensity score matching to address internal validity and reduce confounding variables, several limitations must be acknowledged. First, as a retrospective analysis using the TriNetX database, the study can demonstrate associations but cannot establish causal relationships between NAC reconstruction and mental health outcomes. Second, reliance on ICD-10, CPT, and ATC codes, while standardized, may not fully capture the clinical nuances of each reconstructive procedure or the complexity of patients’ mental health diagnoses (eg, differing outcomes of different types of NAC reconstruction^14-18^). Third, although propensity score matching was utilized to balance measured variables between cohorts, residual covariates from unmeasured factors, such as surgeon preferences, exact surgical techniques,^14,16,18^ or patient motivation for NAC completion, may still influence the results. Fourth, we were unable to control the precise timing between mastectomy, breast reconstruction, and subsequent NAC reconstruction. Such granularity is not consistently available in the database, and restricting the cohort to patients with complete temporal data would have significantly reduced the sample size and statistical power. Fifth, variability in documentation practices across institutions and providers, as well as potential underreporting or miscoding of mental health conditions, may have introduced bias or led to outcome misclassification. Sixth, the database does not capture patient-reported outcomes, limiting our ability to assess subjective satisfaction, perceived body image, or sexual well-being, which are factors known to influence mental health after breast reconstruction. Lastly, we were unable to account for cancer staging, as specific ICD-10 codes for tumor stage are not available within the TriNetX database.

Future studies should prioritize prospective, longitudinal designs that incorporate validated tools such as the BREAST-Q to acquire more granular data on patient opinions, satisfaction scores, and psychological well-being. Comparing NAC and non-NAC cohorts using standardized patient-reported outcome measures would provide deeper insight into the interplay between surgical choices and psychosocial recovery. Our findings contribute to the growing body of evidence suggesting that NAC reconstruction may serve as a marker of stronger baseline psychological well-being and highlight the importance of reconstructive planning that integrates both surgical and psychosocial considerations.

## CONCLUSION

In this large, multi-institutional retrospective cohort study, NAC reconstruction following mastectomy was associated with a lower incidence of anxiety, adjustment disorder, substance use disorder, and antidepressant use across multiple postoperative timepoints compared with reconstruction without NAC. These findings suggest potential psychosocial benefits for patients who complete their breast reconstruction with NAC restoration. However, premorbid psychological factors may influence postmastectomy mental health outcomes. Patients who pursue NAC reconstruction may already demonstrate stronger baseline psychological resilience, making the decision to undergo NAC reconstruction both a marker and potential contributor to improved postoperative mental well-being. These results highlight the importance of addressing both surgical and psychosocial factors during patient counseling and shared decision-making. Future prospective studies incorporating validated patient-reported outcome measures, such as the BREAST-Q, are warranted to clarify causal directionality and optimize patient-centered reconstructive care.
